# Population structure of a vector of human diseases: *Aedes aegypti* in its ancestral range, Africa

**DOI:** 10.1002/ece3.4278

**Published:** 2018-07-13

**Authors:** Panayiota Kotsakiozi, Benjamin R. Evans, Andrea Gloria‐Soria, Basile Kamgang, Martin Mayanja, Julius Lutwama, Gilbert Le Goff, Diego Ayala, Christophe Paupy, Athanase Badolo, Joao Pinto, Carla A. Sousa, Arlete D. Troco, Jeffrey R. Powell

**Affiliations:** ^1^ Yale University New Haven Connecticut; ^2^ Centre for Research in Infectious Diseases P.O. Box 13591 Yaoundé Cameroon; ^3^ Uganda Virus Research Institute Entebbe Uganda; ^4^ MIVEGEC Laboratory (UMR IRD 224‐5290 CNRS‐UM) Institut de Recherche pour le développement (IRD) Montpellier France; ^5^ IRD La Réunion‐GIP CYROI La Réunion France; ^6^ Centre International de Recherches Médicales de Franceville (CIRMF) Franceville Gabon; ^7^ Laboratoire d'Entomologie Fondamentale et Appliquée Université Ouaga 1 Pr Joseph KI‐ZERBO 03 BP 7021 Ouagadougou 03 Burkina Faso; ^8^ Global Health and Tropical Medicine, GHTM Instituto de Higiene e Medicina Tropical, IHMT Universidade Nova de Lisboa, UNL Lisbon Portugal; ^9^ Programa Nacional de Controle da Malária Direcção Nacional de Saúde Pública Ministério da Saúde Luanda Angola; ^10^ Present address: The Connecticut Agricultural Experiment Station New Haven Connecticut

**Keywords:** *Aedes aegypti*, Africa, genetics, migration, population structure, SNP‐chip

## Abstract

*Aedes aegypti*, the major vector of dengue, yellow fever, chikungunya, and Zika viruses, remains of great medical and public health concern. There is little doubt that the ancestral home of the species is Africa. This mosquito invaded the New World 400‐500 years ago and later, Asia. However, little is known about the genetic structure and history of *Ae. aegypti* across Africa, as well as the possible origin(s) of the New World invasion. Here, we use ~17,000 genome‐wide single nucleotide polymorphisms (SNPs) to characterize a heretofore undocumented complex picture of this mosquito across its ancestral range in Africa. We find signatures of human‐assisted migrations, connectivity across long distances in sylvan populations, and of local admixture between domestic and sylvan populations. Finally, through a phylogenetic analysis combined with the genetic structure analyses, we suggest West Africa and especially Angola as the source of the New World's invasion, a scenario that fits well with the historic record of 16th‐century slave trade between Africa and Americas.

## 1 INTRODUCTION

The mosquito *Aedes aegypti* is the major vector of diseases such as dengue, chikungunya, yellow fever, and Zika, that have plagued humanity for centuries and remain threats to millions of people worldwide. It is an invasive species with patterns of global migration that continue today (Powell, 2016).

There is little doubt that the ancestral range of the species is Africa. The ancestral form has been given the subspecies name *Ae. aegypti formosus* (Aaf), a dark mosquito breeding in tree holes and preferring blood meals from nonhuman wildlife (Lounibos, 1981; Powell & Tabachnick, 2013; Tabachnick, 1991). Aaf can be found today in Africa in its original sylvan habitats (larvae in tree holes and rock holes), as well as in cities and peridomestic habitats (e.g., villages, transient human dwellings, and their surroundings). The paler form, or subspecies *Ae. aegypti aegypti* (Aaa), is a “domestic” mosquito, breeding in human‐generated containers and preferring humans for blood meals (McBride et al., 2014). It is this form that during the last 400‐500 years colonized much of the world's tropics and subtropics with the help of human movement and trade (Powell, 2016; Powell & Tabachnick, 2013), causing some of the largest outbreaks of mosquito‐borne diseases, most recently the Zika outbreak (Centers for Disease Control and Prevention 2016).

While populations outside Africa (largely conforming to Aaa) have been well‐studied and strong genetic structure among and within continents have been documented [e.g., (Bosio et al., 2005; Bracco, Capurro, Lourenço‐de‐Oliveira, & Sallum, 2007; Brown et al., 2011, 2014; Gloria‐Soria et al., 2016; Gonçalves da Silva et al., 2012; Kotsakiozi, Gloria‐Soria, Schaffner, Robert, & Powell, 2018; Kotsakiozi, Gloria‐Soria et al., 2017; Mousson, Dauga, Garrigues, & Schaffner, 2005; Pless et al., 2017; Rašić et al., 2015; Scarpassa, Cardoza, & Cardoso, 2008)], the ancestral populations in Africa have been understudied. Even *Ae. aegypti’*s range in Africa is poorly known due to insufficient records of the species (Weetman et al., 2018). Additionally, the types of genetic markers (e.g., allozymes, mtDNA, and microsatellites) used in previous studies have been unable to provide much insight into the genetic structure in this ancestral region although Bennett et al. (2016) did provide genetic resolution using DNA sequences, discussed later in the context of our findings. More specifically, although it seems that there are at least two major genetic clusters of *Ae. aegypti formosus* in East and West Africa, further resolution has proven difficult with allozymes or microsatellites (Brown et al., 2011; Gloria‐Soria et al., 2016; Moore et al., 2013).

Understanding the genetic structure of *Ae. aegypti* within Africa in high resolution and predicting the invasion dynamics and gene flow among populations can be very informative and helpful to control and predict future outbreaks of diseases they transmit. In Africa alone, more than 800 million people (~70% of the African population) are at risk for at least one of the diseases transmitted by this species (Weetman et al., 2018). Contrary to the traditional view that African Aaf is less competent for flavivirus transmission than Aaa outside Africa (Bosio, Beaty, & Black, 1998; Tabachnick et al., 1985), there is increasing evidence that the vector competence of Aaf varies considerably and is population‐specific, with some African populations being as competent as those outside Africa (Diallo et al., 2008; Dickson, Sanchez‐Vargas, Sylla, Fleming, & Black, 2014; Vazeille et al., 2013).

To address this challenge, we leverage a high‐throughput species‐specific genotyping single nucleotide polymorphism (SNP) chip (Evans et al., 2015). Dense genomic sampling of SNPs is extremely powerful for high‐resolution analysis of historical biogeography and invasion dynamics [e.g., in the study of *Aedes* species (Brown et al., 2014; Kotsakiozi, Richardson et al., 2017; Rašić, Filipović, Weeks, & Hoffmann, 2014)]. The goals of this work were to (a) study the genetic structure of *Ae. aegypti* populations within Africa, (b) estimate the genetic diversity and differentiation among African populations and compare them with Aaa populations outside of Africa, and (c) identify the possible source(s) of the New World and Asia invasion.

*Note on nomenclature*: The subspecies designations *Aedes aegypti formosus* and *Ae. aegypti aegypti* were formally recognized by Mattingly (1957) with the former being a darker colored mosquito in African forests, while the latter are lighter colored with white abdominal scales found in human habitats primarily outside Africa. While generally, collections of *Ae. aegypti* in Africa correspond to subspecies *Ae. aegypti formosus,* there are exceptions with some back migration of Aaa to Africa (particularly in East Africa) and mixed populations in West Africa which may represent the initial differentiation of Aaa (Crawford et al., 2017). Here, we use Aaf as shorthand to refer to populations in continental Africa and Aaa to refer to populations outside Africa with the explicit recognition these names are not clear‐cut especially in Africa. In Table 1, we designate the ecological setting where the samples from Africa were taken to explicitly recognize the ecological diversity occupied by this species in Africa.

**Table 1:** Population information for the *Aedes aegypti* samples used in this study

Continent	Region	Country/island	Locality (abbreviation)	Type	Samples	SNPs	latitude	longitude
Africa	West Africa	Angola	Luanda (Ang)	Domestic	12	16,906	−9.76667	14.26667
		Burkina Faso	Burkina Faso (BF)	Domestic	12	16,855	12.2383	−1.5616
		Cameroon	Yaounde Mokolo (YAOMO)	Domestic	7	16,845	3.87275	11.5012
		Cameroon	Yaounde MvogAda (YAOMV)	Domestic	8	16,804	3.86275	11.5259
		Cameroon	Yaounde Center (CAM)	Domestic	12	16,877	3.866667	11.5167
		Cameroon	Yaounde Forest (YAOF)	Sylvan	8	16,758	3.87601	11.3761
		Cameroon	Yaounde Village (YAOV)	Peridomestic	8	16,795	3.86076	11.3937
		Cameroon	Buffalo camp (CamD)	Peridomestic	10	16,853	8.371057	13.866
		Gabon	Franceville (GB)	Domestic	12	16,797	−1.63324	13.583
		Gabon	Lope Forest (GB_F)	Sylvan	12	16,801	−0.37896	11.5274
		Gabon	Lope Village (GB_V)	Peridomestic	12	16,701	−0.37896	11.5274
		Senegal	Sedhiou (Sedh)	Peridomestic	12	16,866	14.183	−12.717
		Senegal	Goudiry (Goud)	Peridomestic	12	16,903	12.707	−15.5552
	East Africa	South Africa	Johannesburg (AFS)	Domestic	9	16,777	27.9006	−25.9904
		Uganda	Lunyo (Lun)	Peridomestic	12	16,859	0.3267	33.8936
		Uganda	Zika village (ZIKA)	Peridomestic	14	16,811	0.12745	32.5313
		Kenya	Kaya Forest (KEN)	Sylvan	8	16,861	−3.93194	39.5961
		Kenya	Kahawa Sukari (KS)	Peridomestic	8	16,874	−1.19451	36.9456
		Kenya	Nairobi (NBO)	Domestic	8	16,702	−1.2833	36.8167
		Reunion island	Reunion Island (RI)	Domestic	12	14,499	−20.1818	57.5171
		Mauritius island	*Aedes mascarensis* (Masc)	Outgroup	4	13,286	−20.1668	57.5147
Asia		Australia	Cairns (Cairns)	Aaa	12	16,990	−16.817	145.686
		Georgia	Georgia (Georgia)	Aaa	10	16,927	41.9614	43.3624
		Philippines	Philippines (BBG)	Aaa	8	17,005	10.2833	123.947
		Tahiti	Tahiti (FP)	Aaa	12	17,000	−17.531	−149.56
		Vietnam	Ho Chi Minh (HCM)	Aaa	12	16,976	10.8032	106.695
New World		Brazil	Macapà (AJM)	Aaa	12	16,935	0.03542	−51.071
		Caribbean	Dominica (Dom)	Aaa	12	16,938	15.59166	−61.4111
		Colombia	Cali (Cali)	Aaa	12	17,012	3.43894	−76.516
		Siquirres	Costa Rica (CR)	Aaa	6	16,394	9.93848	−84.095
		Mexico	Chetumal (CheDC) lab strain	Aaa	8	16,997		

Note

For each population, the sampling locality (with abbreviation), the ecological setting where sampled, the number of mosquitoes analyzed, the average number of SNPs obtained, and location in latitude/longitude for the samples are presented.

## 2 METHODS

### 2.1 Mosquito samples, DNA extraction, and genotype process

**Figure 1:**
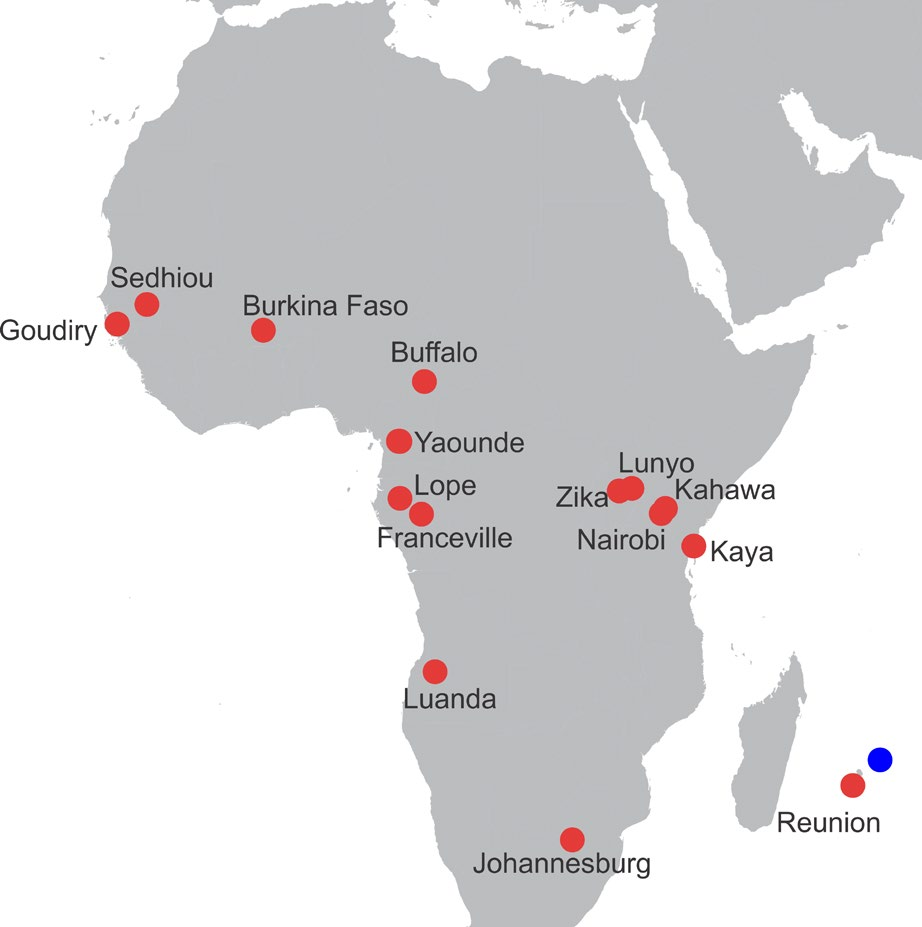
Locations of *Ae. aegypti* sampled from mainland Africa and Reunion Island. Two of the sampling localities, Yaounde and Lope, include 5 and 2 sampling sites, respectively. The multiple sampling points in these localities are less than 3 km apart. The blue sampling site represents *Ae. mascarensis* used as outgroup

We sampled 20 populations of *Aedes aegypti* originating from continental Africa and nearby Reunion Island (Figure 1, Table 1) covering a large part of the Aaf distribution. We also used 10 previously studied populations (Gloria‐Soria et al., 2018; Kotsakiozi et al., 2018) of Aaa originating from the New World and Asia (Table 1). *Aedes mascarensis* from the island of Mauritius was used as an outgroup; this species is very closely related to *Ae. aegypti* being able to form viable hybrids (Hartberg & Craig, 1970), but genetically distinct (Brown et al., 2014). Samples were either larvae preserved in 70%–90% ethanol, collected from multiple breeding sites per sampling locality, or eggs collected from multiple ovitraps set up at various locations. Eggs were reared to larvae or adults in standard laboratory conditions. DNA was extracted with Qiagen DNeasy blood/tissue kit using the standard kit protocol with an additional step of adding 4ul of RNAase A to each sample. Approximately 200 ng of genomic DNA from individual mosquitoes were placed in 95 wells of a 96‐well plate, with one distilled water control. Plates were sent to the Functional Genomics Core at the University of North Carolina, Chapel Hill, for hybridization. Data files sent to Yale University were processed with the Axiom Analysis Suite v.3.1. (Affymetrix, Santa Clara, CA) to call the genotypes. We genotyped 7‐14 individuals per population (Table 1) to avoid large differences in sampling size between populations that can obscure the subsequent genetic structure analyses [for details on the effect of uneven sample size on genetic structure analyses, see (Puechmaille, 2016; Wang, 2017)]. This sample size is considered adequate for the purposes of the study, given the large number of SNPs assayed, the very low percentage of missing data (Evans et al., 2015), and the expected differentiation among populations (Gloria‐Soria et al., 2016) estimated from previous studies [for details on the sampling size discussion and examples of using similar sampling size, see (Brown et al., 2014; Nazareno, Bemmels, Dick, & Lohmann, 2017; Patterson, Price, & Reich, 2006; Puckett et al., 2016)]. Here, we report results on a total, 315 mosquitoes (208 Aaf, 104 Aaa, and four *Ae. mascarensis*) genotyped using the Axiom_aegypti1 genotyping array (Evans et al., 2015). A total of 27,674 loci were included in the Axiom_aegypti1 SNP‐Chip (overall genotyping rate 97.1%) unambiguously genotyped on the chip and passed the tests for conformance to being inherited as single‐copy Mendelian variants (Evans et al., 2015).

### 2.2 Genetic structure analyses

From the 27,674 validated loci available on the *Ae. aegypti* SNP‐chip, a subset of 20,117 were variable in our dataset of 315 samples (hereafter referred to as *broad dataset*) including both Aaf and Aaa samples (as well as *Ae. mascarensis*). We further filtered this dataset eliminating highly linked loci using the—indep option (SNP window size = 500, window shift size = 50, variance inflation factor = 2) of plink (Purcell et al., 2007), so the final filtered dataset consisted of 17,069 SNPs. This allows us to use analytical procedures that assume independence across loci. The average percentage of missing data per sample in this dataset was 2%, and details on the average number of SNPs used per population are provided in Table 1.

Population genetic structure was evaluated using the Bayesian clustering method implemented in the software fastSTRUCTURE (Raj, Stephens, & Pritchard, 2014). We performed 10 independent runs, and the results were summarized and plotted using the online version of CLUMPAK (Kopelman, Mayzel, Jakobsson, Rosenberg, & Mayrose, 2015).

To complement the genetic structure analysis, we performed principal component analysis (PCA) and discriminant analysis of principal components (DAPC), using the R packages LEA (Frichot & Francois, 2015) and ADEGENET (Jombart, 2008), respectively, in R v.3.4.4 (R Core Team 2018). In DAPC analysis, the raw data is first transformed through a PCA and then a discriminant analysis (DA) is performed on the retained principal components (PCs). Thus, DAPC analysis can provide an efficient description of the genetic clusters present in the dataset using a few synthetic variables (discriminant functions). These variables are linear combinations of the original variables (raw data) that maximize the between‐group variance and minimize the within‐group variance.

### 2.3 Genetic diversity and differentiation

Pairwise genetic distances (Fst) between all pairs of populations and their significance (significance level of 0.05) were calculated in Arlequin v3.5.2.2 (Chapuis & Estoup, 2007), using 1,000 permutations.

The partitioning of the genomic variation among and within populations was evaluated through a hierarchical analysis of molecular variance, AMOVA (Excoffier, Smouse, & Quattro, 1992), as implemented in Arlequin v.3.5.2.2, using 1,000 permutations. For this analysis, we excluded *Ae. mascarensis* because it is used as outgroup and Reunion samples because of high differentiation (see Results section). The partitioning of the genomic variation was evaluated in the following levels: 1) Africa/outside Africa, 2) West Africa/East Africa, 3) domestic/peridomestic/sylvan populations, and 4) between the African countries. Details on the grouping for the AMOVA analyses are provided in Table 1.

### 2.4 Isolation by distance

To assess the significance of correlation between geographic (Euclidean distance) and genetic distance matrices, for all the African populations, we performed a Mantel test with 999 permutations using the “ade4” package in R v.3.4.4 (R Core Team 2018).

### 2.5 Phylogenetic relationships

To infer the evolutionary relationships among the populations, we used a maximum likelihood (ML) analysis, as implemented in RaxML (Stamatakis, 2014), using 1,000 bootstraps and the GTR model of evolution along with the CAT model of rate heterogeneity. For the runs, we used the string “ASC” to apply an ascertainment bias correction to the likelihood calculations, and the standard correction by Lewis (2001) when only variable sites are included in the dataset. For the phylogenetic analysis, we excluded SNPs that were identified as outliers (qvalues < 0.01) using the pcadapt R package (Luu, Bazin, & Blum, 2017), because such SNPs might be under selection. We also randomly sampled two individuals per population for each African samples, and we included two *Ae. mascarensis* individuals as an outgroup and six samples of Aaa outside Africa (two each from South America, North America, Asia) to confirm the distinctiveness of the Aaa lineage from all the African lineages (Bennett et al., 2016; Brown et al., 2014). The final SNP dataset used for the phylogenetic analysis consisted of 12,471 SNPs.

## 3 RESULTS

### 3.1 Genetic structure analyses

**Figure 2:**
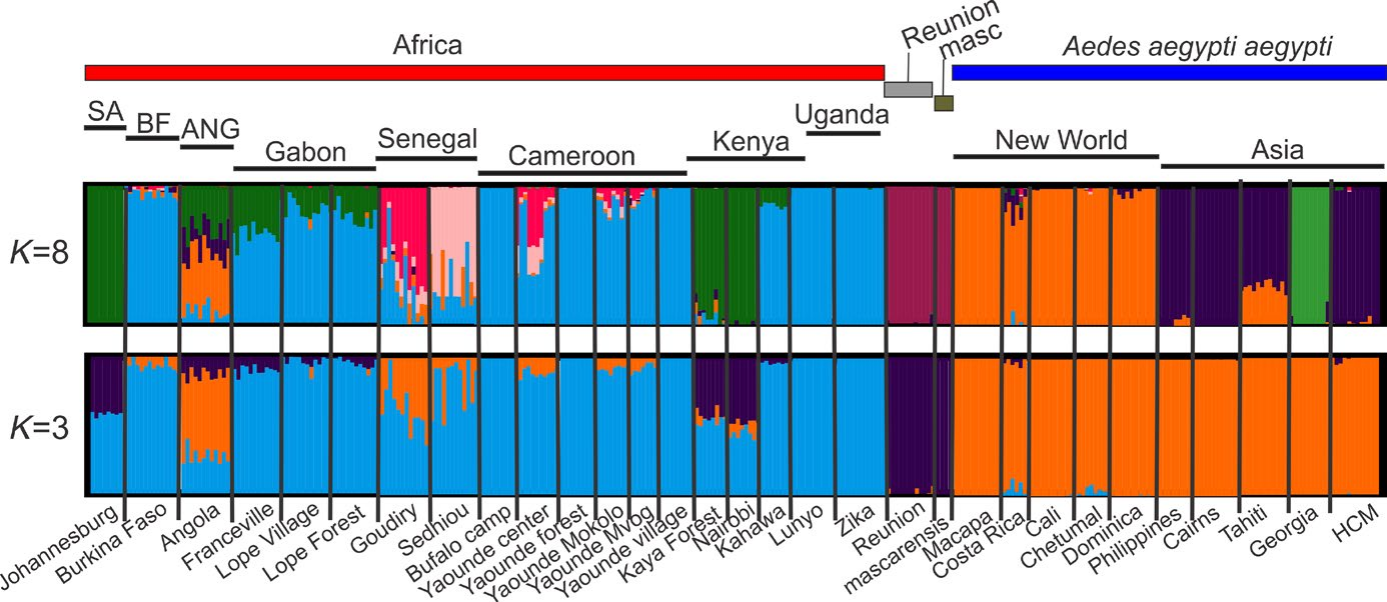
STRUCTURE bar plots for all *Ae. aegypti* populations and *Ae. mascarensis*. Population names are reported on the *x*‐axis. The *y*‐axis reports the probability of each individual (*Q*‐value) assigned to one of the genetic groups identified by fastSTRUCTURE, which are represented by different colors. Each bar represents an individual. Individuals with 100% assignment to one group are identified by a single color. Individuals with mixed ancestry are represented by bars with different percentages of colors. The thick black lines within the plots indicate population limits. Abbreviations: SA: South Africa, BF: Burkina Faso, ANG: Angola, masc: *Ae. mascarensis*

**Figure 3:**
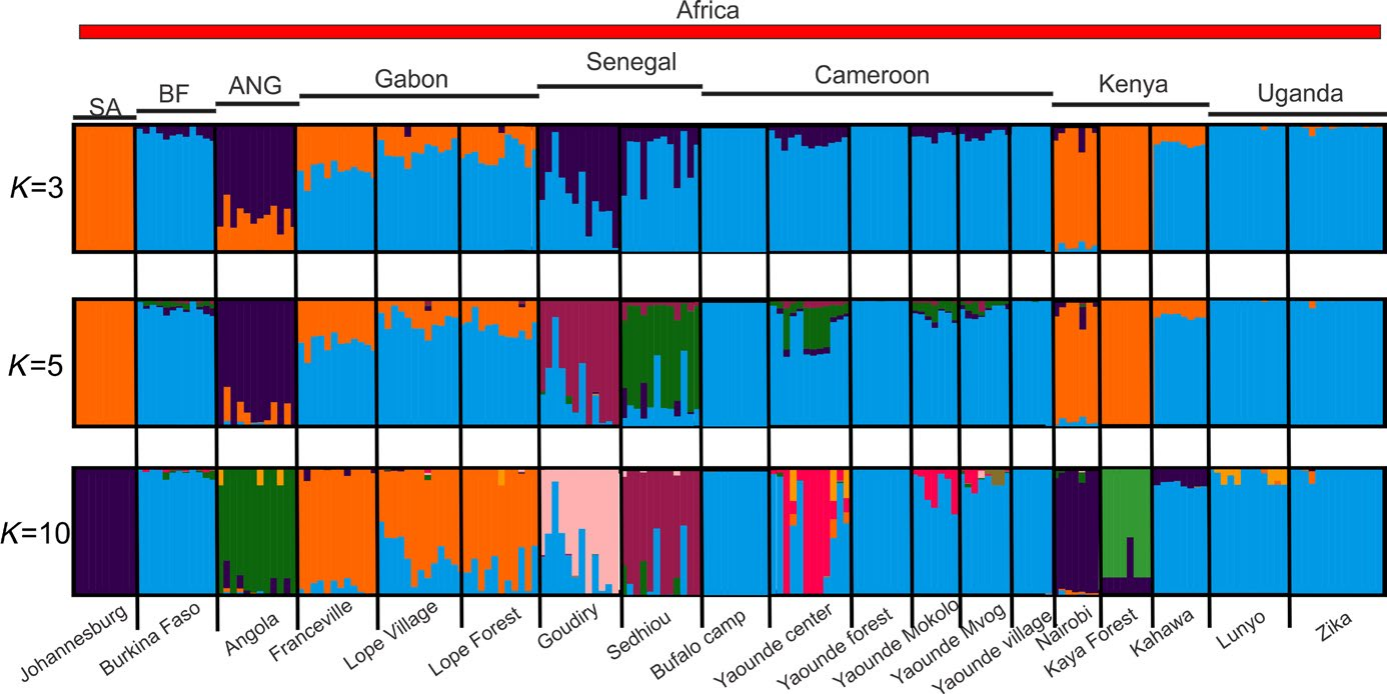
STRUCTURE bar plots for all African *Ae. aegypti* populations. Population names are reported on the *x*‐axis. For details, see legend of Figure2

The results of the fastSTRUCTURE analyses on the *broad* (all samples) and the *African dataset* are shown in Figure 2 and Figure 3, respectively. The structure analysis on the *broad dataset* (Figure 2, K = 3) supported that all the African populations used in this study are distinct from all the Aaa populations outside Africa, with only three samples (Goudiry and Sedihou, Senegal and Angola) showing significant admixture. For these three populations, the average *Q* values (for K = 3; Figure 2) toward the Aaa cluster equal to 0.18 for Sedhiou, 0.38 for Goudiry, and 0.60 for Angola. Additionally, Angola is the only population that retains the admixed pattern for K = 8 as well, showing an average *Q* value of 0.42 toward the New World Aaa cluster (K = 8; orange) and 0.31 toward the South Africa‐Kenya cluster (K = 8; green).

Interestingly, the Indian Ocean island samples, Reunion and *Ae. mascarensis* from Mauritius, cluster together. Three additional African populations (Figure 2; K = 3; Johannesburg, Kaya Forest, and Nairobi) seem to be admixed with the Reunion cluster (*Q* values; 0.44, 0.49, and 0.43 for AFS, KEN, and NBO, respectively).

Focusing on the continental African dataset (Figure 3), it becomes evident that (a) Uganda, Burkina Faso, and Cameroon populations cluster together with Gabon being fairly close although distinct at K = 10, (b) Angola forms a separate group, (c) the three populations from Gabon are indistinguishable from each other, (d) South Africa clusters with Nairobi and (e) two populations from Senegal are well differentiated from each other as are populations from Kenya (three populations form three clusters; K = 10).

**Figure 4:**
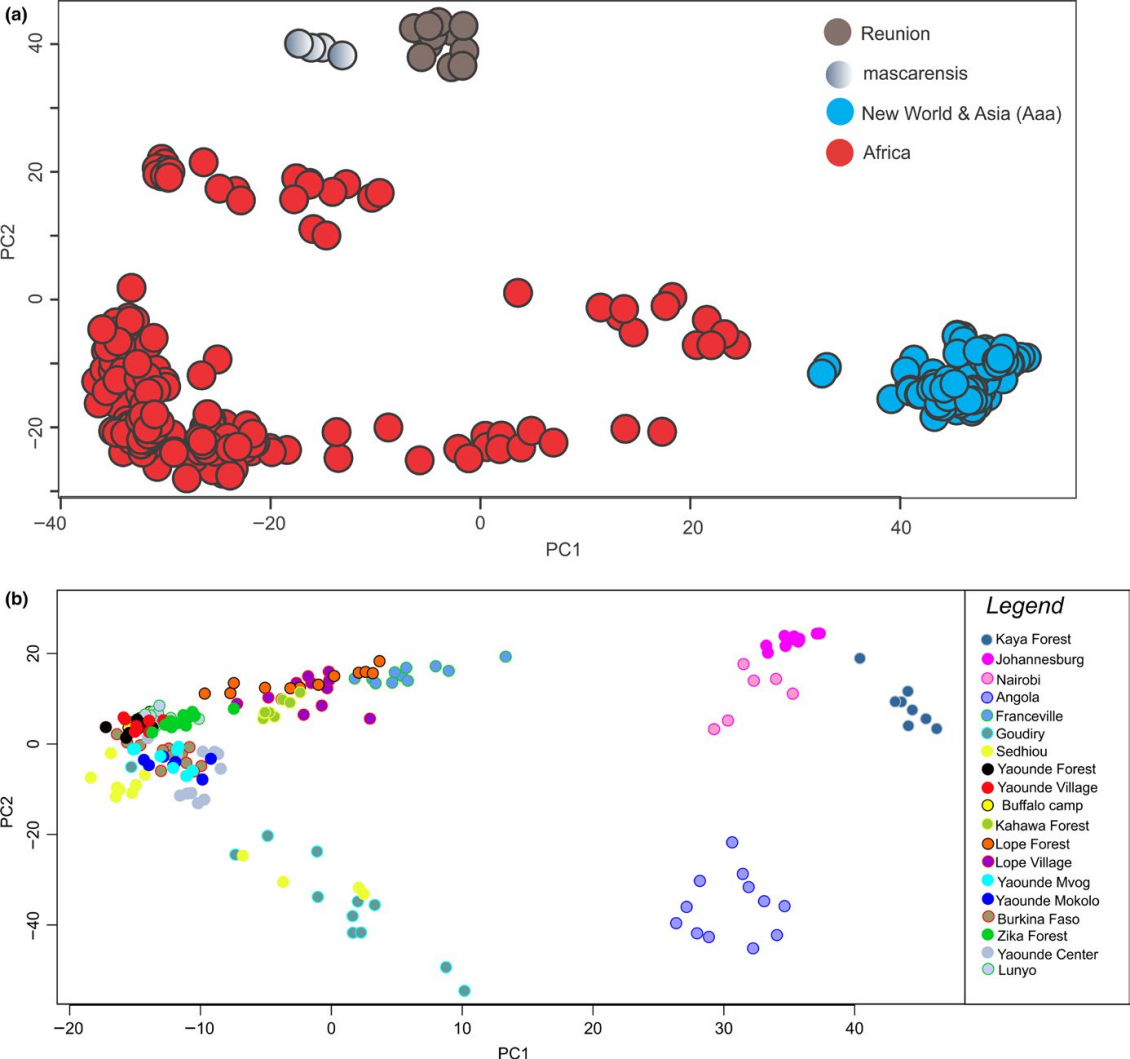
Principal components analysis (PCA) on the *broad dataset* including all the *Ae. aegypti* populations as well as the *Ae. mascarensis* (a) and including only the African populations (b). PCA implemented and plotted in LEA R package, presenting the projection of all individual mosquitoes on the first two PCs. Populations originated from different regions are presented with different colors as shown in the inset

Principal component analyses on both datasets confirmed the results obtained from fastSTRUCTURE. Specifically, when using the *broad* dataset (Figure 4a), the differentiation between Aaa and Aaf is clear as well as the genetic uniqueness of *Ae. mascarensis*/Reunion populations. The PCA for only continental African samples (Figure 4b) generally mirrors what fastSTRUCTURE revealed (Figure 3).

**Figure 5:**
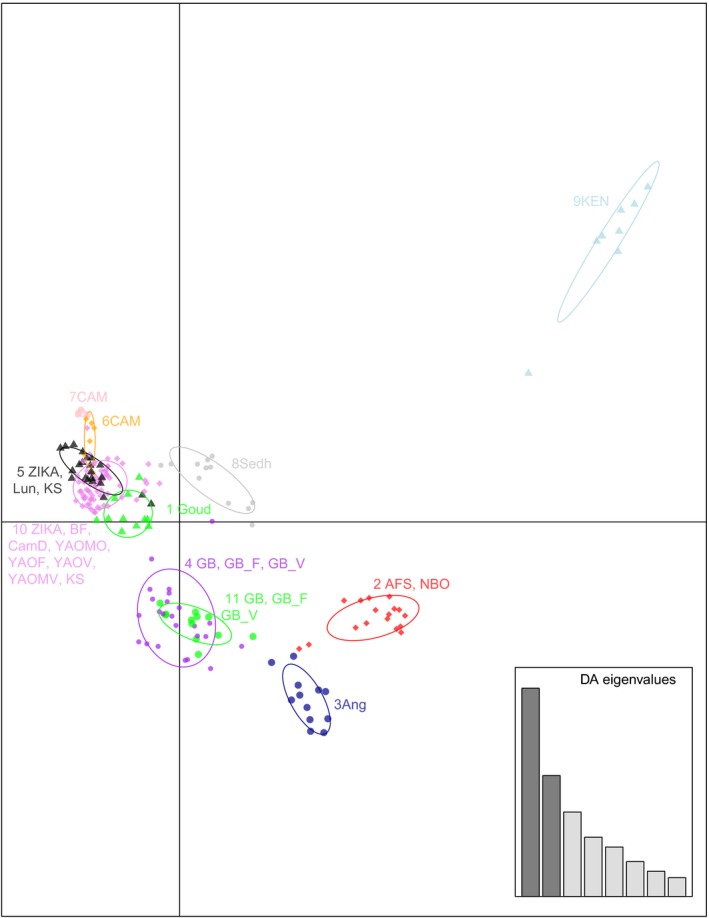
Discriminant analysis of principal components (DAPC) for the African populations as implemented and plotted in “adegenet” R package. The graph represents the individuals as dots and the groups as inertia ellipses. A bar plot of eigenvalues for the discriminant analysis (DA eigenvalues) is displayed in the inset. The bars in the inset represent the number of discriminant functions retained in the analysis, the first two of which are used in the plot. Population codes are as shown in Table 1

DAPC analysis on the continental African samples (Figure 5) with 11 groups identified by the Bayesian information criterion (BIC) generally coincides with the K = 10 results of the fastSTRUCTURE analysis (Figure 3). In particular, in DAPC, South Africa clusters together with Nairobi (group 2, red) while Angola, Kaya forest, Sedhiou, and Goudiry each form separate groups (groups 3, 9, 8, and 1, respectively). Although some of the Gabon samples form a separate group from the remaining Gabon individuals, their clouds overlap (groups 4 and 11). Similarly, samples from Uganda, Burkina Faso, and Cameroon form four overlapping DAPC groups.

### 3.2 Genetic diversity and differentiation

Table 2 shows the pairwise Fst values between the African populations. All pairwise Fst estimations were significant at significance level 0.05. The mean genetic differentiation between Africa and New World is 0.32 and somewhat higher between Africa and Asia, 0.35. The Reunion sample, while technically coming from Africa, is as differentiated as Africa/outside Africa samples, average Fst of 0.33.

**Table 2:** Analyses of molecular variance (AMOVA) as implemented in Arlequin

Groups	Source of variation	*df*	Percentage of variation (%)
Africa/out of Africa	Among groups	1	20.79
	Within groups	28	13
	Within populations	592	66.21
West Africa/East Africa	Among groups	1	1.89
	Within groups	17	12.87
	Within populations	371	85.23
BF/Kenya/Uganda/Angola/SA/Cameroon/Gabon/Senegal	Among groups	7	6.37
	Within groups	11	8.13
	Within populations	371	85.5
Domestic/Peridomestic/Sylvan	Among groups	2	0.05
	Within groups	13	13.82
	Within populations	371	86.13

Notes

Populations are divided into groups as shown in Table 1.

BF: Burkina Faso; *df*: degrees of freedom; SA: South Africa.

The results of the analysis of AMOVA are presented in Table 3. The majority of the genetic variation in our dataset, regardless of the grouping, is within the populations. However, a great deal of variation (~20%) exists between groups in the first AMOVA analysis (Africa/outside Africa) confirming the pattern in Figure 2 and Figure 4. Also, the results of the third AMOVA analysis are consistent with the patterns observed in both fastSTRUCTURE (Figure 3; K = 10) and DAPC (Figure 5).

**Table 3:** Population Differentiation

	1	2	3	4	5	6	7	8	9	10	11	12	13	14	15	16	17	18
1: Buffalo camp																		
2: Yaounde Mokolo	0.09																	
3: Yaounde Mvog	0.05	0.07																
4: Yaounde Center	0.08	0.07	0.06															
5: Yaounde Forest	0.03	0.08	0.04	0.08														
6: Yaounde Village	0.02	0.08	0.04	0.07	0.01													
7: Burkina Faso	0.03	0.07	0.04	0.07	0.04	0.03												
8: Luanda Angola	0.20	0.19	0.17	0.19	0.20	0.19	0.18											
9: Goudiry	0.15	0.16	0.13	0.15	0.14	0.13	0.13	0.15										
10: Sedhiou	0.13	0.15	0.12	0.15	0.11	0.11	0.11	0.20	0.16									
11: Johannesburg	0.22	0.26	0.23	0.24	0.22	0.21	0.22	0.22	0.27	0.27								
12: Kahawa Sukari	0.06	0.11	0.07	0.10	0.06	0.05	0.07	0.19	0.16	0.14	0.18							
13: Kaya Forest	0.28	0.30	0.27	0.28	0.28	0.27	0.26	0.23	0.29	0.31	0.22	0.25						
14: Nairobi	0.18	0.20	0.19	0.20	0.18	0.17	0.17	0.17	0.22	0.23	0.07	0.15	0.20					
15: Lope Forest	0.06	0.11	0.08	0.11	0.06	0.05	0.07	0.20	0.17	0.15	0.16	0.07	0.25	0.14				
16: Lope Village	0.05	0.09	0.07	0.09	0.05	0.04	0.06	0.18	0.15	0.14	0.15	0.06	0.23	0.13	0.01			
17: Franceville	0.08	0.13	0.10	0.12	0.09	0.08	0.09	0.19	0.19	0.17	0.13	0.08	0.23	0.12	0.05	0.04		
18: Lunyo	0.07	0.11	0.08	0.11	0.06	0.06	0.07	0.21	0.17	0.15	0.23	0.08	0.29	0.20	0.10	0.08	0.11	
19: Zika	0.04	0.09	0.06	0.09	0.04	0.04	0.05	0.20	0.15	0.13	0.20	0.05	0.26	0.17	0.07	0.06	0.09	0.05

Note

Pairwise Fst values between African populations of *Ae. aegypti* as estimated based on the panel of ~17K SNPs, using Arlequin. All values are significant at significance level 0.05.

**Figure 6:**
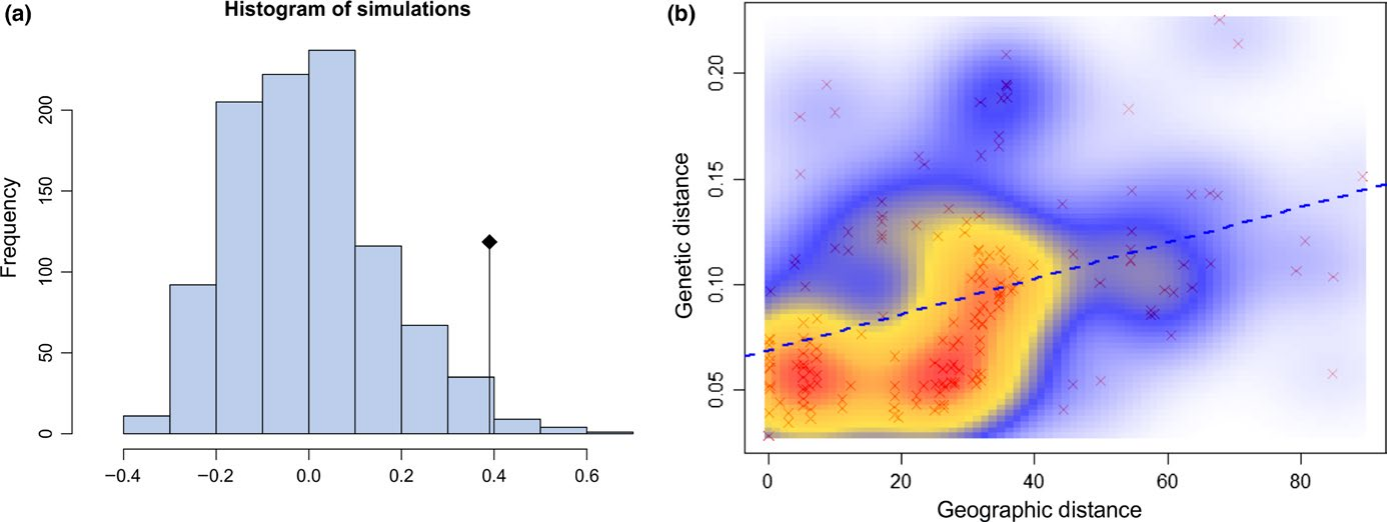
Isolation‐by‐distance plots for all pairs of populations from continental Africa. Statistical significance was evaluated through a Mantel test as implemented in the “ade4” R package. The original value of the correlation between the two matrices (geographic distance and genetic distance) is represented by a dot, while the histogram (a) represents the permutated values assuming the absence of spatial structure. Significant spatial structure results in the original value being out of the reference distribution. The correlation between geographic and genetic distance was plotted using the R package “MASS.” The scatterplot (b) shows one single consistent cloud of points. The colored gradient from light blue to red indicates the density of the points which are also shown as red points in the background of the graph. The blue dashed line represents the regression line between the geographic and genetic distance

Because the results of both the genetic structure and the partitioning of molecular variance analysis suggested isolation by distance, we performed a Mantel test on the Africa dataset to test this hypothesis. The results show marginally significant (p‐value 0.03) signs of isolation by distance (IBD) among the African populations (Figure 6). This is consistent with the findings of Gloria‐Soria et al. (2016), presented in (Figure 4a) where microsatellites displayed IBD, less than in the New World.

### 3.3 Phylogenetic analysis

**Figure 7:**
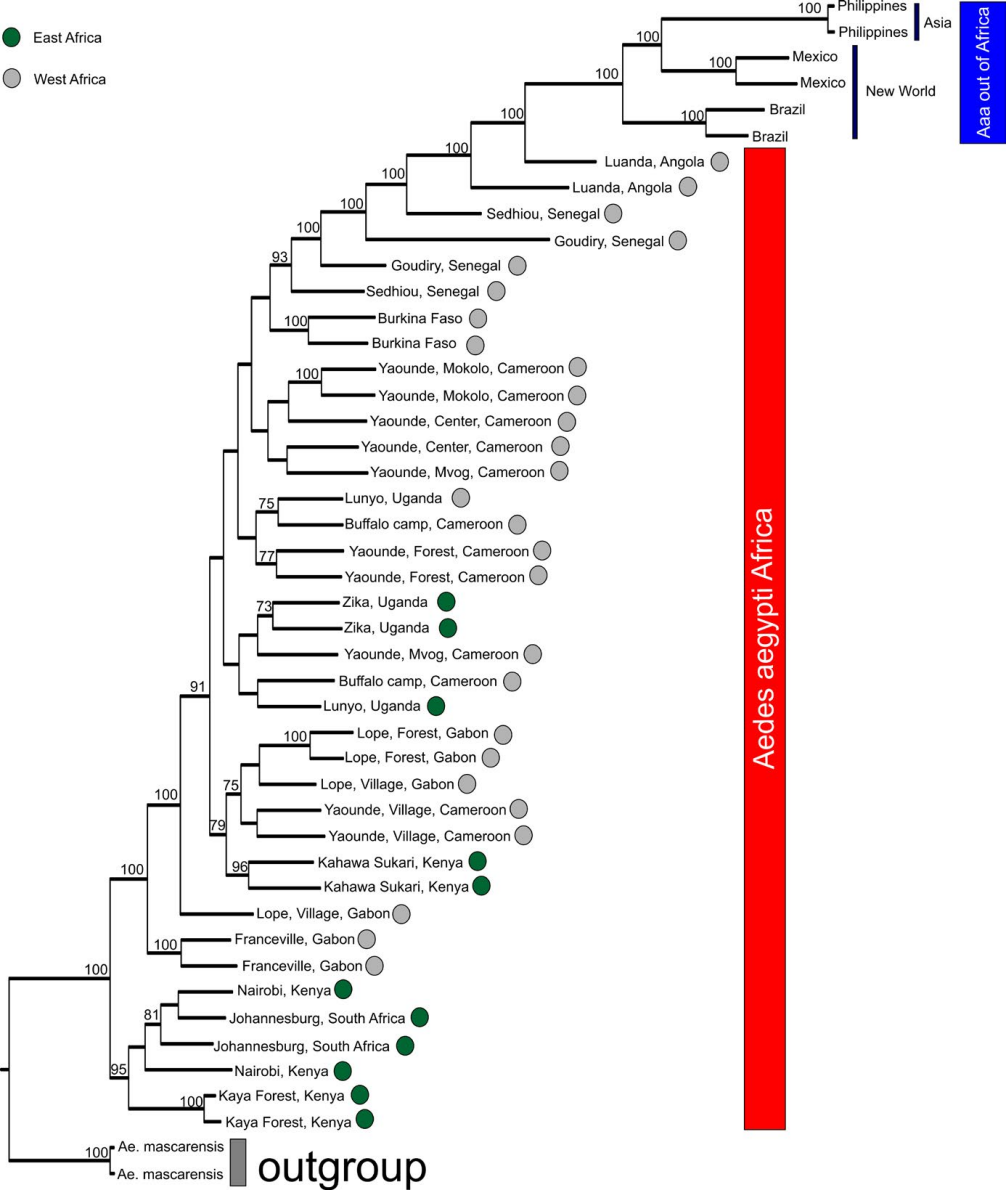
Maximum likelihood (ML) rooted phylogenetic tree re‐constructed using a panel of ~12,000 SNPs. *Ae. mascarensis* was used as an outgroup, and Aaa samples from New World and Asia were used to test the distinctiveness of Aaf and Aaa lineages. Bootstraps are presented on the nodes; values <70 are not shown

The rooted ML phylogenetic tree is presented in Figure 7. All Aaa populations outside Africa form a monophyletic group distinct from all the African Aaf populations. Consistent with their admixture patterns (Figure 2), Senegal and Angola populations are closer related to the Aaa lineage compared with the remaining African populations. The relationships between Cameroon, Gabon, Uganda, and Burkina Faso populations are unresolved. Because the focus here is to resolve patterns in continental Africa, Reunion was not included in the phylogenetic analysis.

## 4 DISCUSSION

Considering the global scale, the SNP‐chip data (Figures 2 and 4a) are consistent with microsatellite, and RAD‐seq studies in showing that *Ae. aegypti* has two major genetic groups. These two groups generally correspond to the described subspecies, *Ae. aegypti formosus* (Aaf) in Africa and *Ae. aegypti aegypti* outside Africa (Aaa) with Aaa being monophyletic (Figure 7) thus implying a single out of Africa event (Brown et al., 2014; Gloria‐Soria et al., 2016).

The population from Reunion Island, however, is exceptional in that it clustered with the *Aedes mascarensis* separately from the Aaf continental populations. Three hypotheses can be proffered for this unexpected distinction of Reunion samples. One is that Reunion *Ae. aegypti* has been introgressing with *Ae. mascarensis* (endemic to Mauritius)*,* given the geographic closeness of the two islands, ~120 km apart. *Ae. aegypti* and *Ae. mascarensis* can hybridize and produce fertile offspring (Hartberg & Craig, 1970). The evident clustering of Reunion with *Ae. mascarensis* (Figure 2) is consistent with this hypothesis. The fact that the three Aaf African populations from East Africa and South Africa (AFS, NBO, KEN) are partially admixed toward the Reunion/*Ae. mascarensis* genotypes (Figure 2; K = 3) also supports the hypothesis of introgression between the two species when geographically close. A second possibility is that Reunion, being ~1200 km from mainland Africa, has been isolated for considerable time, although simple isolation does not address the issue of its genetic closeness to *Ae. mascarensis*. A third possibility we cannot formally dismiss, is that this clustering may be an artifact of biased SNP choice. When the SNP‐chip was designed (Evans et al., 2015), we did not have access to either *Ae. mascarensis* or the Reunion samples, so genetic variation in these populations was not incorporated into the chip design. However, even though *Ae. mascarensis* and Reunion samples genotyped at somewhat fewer loci (Table 1), enough loci (~13–14,000) did genotype to provide reliable data and seems unlikely this could have biased our conclusions.

Considering continental Africa alone, it is clear that ~17,000 SNPs provide better genetic resolution than that provided by 12 microsatellites [e.g., compare Figure 3 here with Figure 3b in Gloria‐Soria et al. (2016)]. Our results confirm the previous findings (Bennett et al., 2016; Brown et al., 2011; Gloria‐Soria et al., 2016) of the existence of two major genetic groups within Africa that roughly correspond to a West‐East differentiation (Figure 7) and at the same time, indicate patterns consistent with both limited migration producing significant isolation by distance as well as long‐distance migration. The clustering of Uganda, Burkina Faso, and Cameroon populations together (Figures 3, 4, and 5) is one striking example of long‐distance gene flow that disrupts the West‐East geographic pattern (Table 2) that had been suggested by previous studies (Bennett et al., 2016; Brown et al., 2011; Moore et al., 2013). This could be due to the fact that the forest habitat typical of ancestral Aaf was continuous across this part of Africa for a long period of time, before human habitation and cutting of forests, allowing enough time in a continuous habitat for even a poor disperser to become relatively genetically homogeneous. Alternatively, the clustering of Kahawa, Kenya, with Cameroon samples (Figures 2, 3, and 7) may imply an old human‐mediated migration across the continent. Bennett et al. (2016) suggested that the Kenya‐Cameroon connection could be due to the populations being once isolated by geographic barriers (e.g., the East African Rift Valley) and then during the Holocene, human migration contributed to mosquitoes migration. Specifically it is known that ~5,000 years ago Bantu farmers moved across the center of Africa from Cameroon to Kenya.

The clearest and most striking example of long‐distance genetic connections is the clustering of two major cities, Nairobi, Kenya (NBO) and Johannesburg, South Africa (AFS) separated by ~3,000 km (Figures 3, 4, and 5), implying long‐distance anthropogenic migration. Nairobi is the only city sampled from the broader Kenya‐Uganda East Africa region which may account for its genetic closeness to the city sampled in South Africa. Commercial trade and human movement between these two major cities are high. The other samples from this region coming from forest or peridomestic habitats (Table 1), do not display such genetic affinities to Johannesburg.

While all evidence point to a single domestication event leading to Aaa outside of Africa, there are secondary, independent domestications taking place within Africa. Genetic patterns suggest that populations in human habitats in Africa today do not have a single origin, and often mix with nearby peridomestic or sylvan populations [as also seen in microsatellite data (Brown et al., 2011)]. While generally domestic collections are closely related to geographically close sylvan or peridomestic collections, the case of Nairobi, discussed above, is an exception and highlights the complex patterns of colonization that occur in Africa.

Using *Ae. mascarensis* as an outgroup, *Aedes aegypti* (sensu *lato*) forms a monophyletic group. Aaa outside Africa (New World and Asia) also forms a monophyletic group implying a single origin (Figure 7). The single out of Africa origin of Aaa has been previously supported by microsatellite (Gloria‐Soria et al., 2016) and RAD‐seq (Brown et al., 2014) data as well as by a combination of five nuclear gene sequences and mtDNA (Bennett et al., 2016). More specifically, Bennett et al. (2016) supported West Africa as most likely origin of Aaa, in agreement with our data (Figure 7).

However, there is a major difference between Bennett et al. (2016) and our results concerning the origin of Aaa in Asia. The ABC analysis of Bennett et al. (2016) favored the New World coming from Asia, although the statistical support for this biogeographic scenario was not strong. Our data here (Figure 7) and elsewhere (Brown et al., 2014; Gloria‐Soria et al., 2016; Kotsakiozi et al., 2018) support with strong statistical power that Asia was derived from the New World.

A recent study (Crawford et al., 2017) based on exome sequence data, suggested that Aaa may have arisen from populations of Aaf in West Africa, specifically from Senegal which was the only West African country sampled in that study. Our data indicate that, while Senegal has some genetic signal typical of Aaa outside Africa, the Angola sample displays an even stronger signal of genetic relatedness to Aaa outside Africa (Figure 2). The population from Angola shows admixed ancestry (*Q* values; 0.42–0.60) toward the New World genotype (Figure 2). Our phylogenetic analysis (Figure 7), including several West African populations (Figure 1), revealed that indeed Senegal samples are phylogenetically closely related to the Aaa, but that Angola is even closer and would be the best candidate for the origin of Aaa.

Using genetic data, the time of origin of Aaa in the New World has been estimated to be ~400‐500 years ago (Crawford et al., 2017; Gloria‐Soria et al., 2016; Kotsakiozi et al., 2018). Yellow fever was first reported in the New World in 1648 (McNeill, 1976) not long after the introduction of *Ae. aegypti* to the New World. This is also the time of the rise of trans‐Atlantic shipping by Europeans. Ships starting their journey in Europe stopped in West Africa to pick up native Africans for the slave trade (Eltis & Richardson, 2010). It is likely that *Ae. aegypti* (as eggs and/or larvae) would have been introduced to those ships and they may have been already semidomesticated in the towns or coastal villages of West Africa (e.g., ovipositing in stored water containers during the prolonged dry periods in West Africa). Thus, these “proto‐Aaa” mosquitoes could survive the long voyage between West Africa and New World. Interestingly, during the early period of slave trade, 1500‐1650, ~70% of the trade was carried out by Portugal (Eltis & Richardson, 2010) with ships that primarily used what is today Angola as their source of slaves (Eltis & Richardson, 2010). An Angolan source of invasion is consistent with the genetic patterns observed (Figure 7).

From a public health perspective, *Ae. aegypti* in Africa has taken on new importance. After decades of low levels, yellow fever has been resurging in Africa (Kraemer et al., 2017). Insecticide resistance and lack of vaccine supplies are doubtlessly contributing to this resurgence. As urban environments continue to encroach on this formerly forest‐adapted mosquito's habitat in Africa, it is clear that Aaf possesses the adaptive flexibility to repeatedly switch to urban breeding. This ongoing active evolution is also an attractive opportunity to study insect adaptations to human habitats, an issue of general importance in a number of medical and agricultural contexts.

## CONFLICT OF INTEREST

None declared.

## AUTHORS’ CONTRIBUTIONS

PK carried out part of the molecular laboratory work, the data analyses, and drafted the manuscript. BE carried out the largest part of the molecular laboratory work and drafted an earlier version of the manuscript. AG‐S carried out part of the molecular work and revised the manuscript critically for important intellectual content. BK, MM, JL, GLG, DA, CP, AB, JP, CAS, and ADT provided samples and revised the manuscript critically for important intellectual content. JRP conceived, designed, and coordinated the study, edited the manuscript, and obtained funds for the research. All authors gave final approval for publication.

## DATA ACCESSIBILITY

The data used are available in vector.base.org under the project ID VBP0000295
